# Supplementary material for: Before hands disappear: Effect of early warning visual feedback method for hand tracking failures in virtual reality
Source: PLoS One. 2025 Jun 10;20(6):e0323796. doi: 10.1371/journal.pone.0323796 (PMC12151392; doi:10.1371/journal.pone.0323796)
Supplement: S1 File — (PDF) [file pone.0323796.s001.pdf]

Detailed Participant Demographics

Participants were recruited through online fliers and emails sent to individuals within the department, faculty, and the university. We also reached out to the public, recruiting participants from outside the university.

4 participants reported using a computer for more than 10 hours a day, primarily for work. 3 participants reported 8-10 hours of daily computer use, 5 participants 6-8 hours, 2 participants 4-6 hours, 2 participants 2-4 hours, and 2 participants reported using a computer for 0-2 hours daily. 14 participants played computer games for 0-2 hours a day, while 3 participants played for 4-6 hours, and 1 participant played for 6-8 hours.

In terms of VR interaction experience, 7 participants had used VR for more than 10 hours, seven participants had 0-2 hours of VR experience, 2 participants had 2-4 hours, 1 participant had 4-6 hours, and 1 participant had 8-10 hours. Among those with more than 10 hours of VR experience, half mentioned using VR for gaming, while the others used it for work.
