# Supplementary material for: Before hands disappear: Effect of early warning visual feedback method for hand tracking failures in virtual reality
Source: PLoS One. 2025 Jun 10;20(6):e0323796. doi: 10.1371/journal.pone.0323796 (PMC12151392; doi:10.1371/journal.pone.0323796)
Supplement: S2 File — (PDF) [file pone.0323796.s002.pdf]

1 ANOVA RESULTS

Below, we provided additional ANOVA results for Task-1 and Task-2. Here, each error condition is analyzed with the Feedback On, Feedback OFF, and benchmark condition.

Table 1. Task-1 Results with ANOVA

	Low Light Intensity	Out of Vision Hands	Self Occlusion
Time	F(2,34)=17.742, p<0.001, $\eta^2 = 0.51$	F(2,34)=101.241, p<0.001, $\eta^2 = 0.856$	F(2,34)=64.340, p<0.001, $\eta^2 = 0.791$
NLT-Fingers	F(2,34)=30.314, p<0.001, $\eta^2 = 0.641$	F(2,34)=31.194, p<0.001, $\eta^2 = 0.647$	F(2,34)=36.124, p<0.001, $\eta^2 = 0.680$
NLT-Hands	F(2,34)=37.454, p<0.001, $\eta^2 = 0.688$	F(2,34)=36.704, p<0.001, $\eta^2 = 0.683$	F(2,34)=74.141, p<0.001, $\eta^2 = 0.6813$
DLT-Hands	F(2,34)=41.314, p<0.001, $\eta^2 = 0.708$	F(2,34)=41.104, p<0.001, $\eta^2 = 0.707$	F(2,34)=5.03, p=0.012, $\eta^2 = 0.228$
N-WP	F(2,34)=1.308, p<0.284, $\eta^2 = 0.071$	F(2,34)=55.602, p<0.001, $\eta^2 = 0.766$	F(2,34)=1.423, p=0.255, $\eta^2 = 0.077$

Table 2. Task-2 Results with ANOVA

	Task-2 Results
Time	F(2,34)=4.407, p<0.01, $\eta^2 = 0.206$
NLT-Fingers	F(2,34)=8.292, p<0.001, $\eta^2 = 0.328$
NLT-Hands	F(2,34)=10.132, p<0.001, $\eta^2 = 0.373$
DLT-Hands	F(2,34)=34.451, p<0.001, $\eta^2 = 0.670$
