# Supplementary material for: Before hands disappear: Effect of early warning visual feedback method for hand tracking failures in virtual reality
Source: PLoS One. 2025 Jun 10;20(6):e0323796. doi: 10.1371/journal.pone.0323796 (PMC12151392; doi:10.1371/journal.pone.0323796)
Supplement: S3 File — (PDF) [file pone.0323796.s003.pdf]

Additional Details about the Procedure

Following the training, participants performed each hand tracking error condition and feedback condition. Each combination was repeated 3 times, taking about 5 minutes on average. After completing 3 repetitions, participants filled out the NASA TLX and SUS questionnaires, which took around 2.5 minutes. Thus, each block of 3 repetitions and questionnaire completion took approximately 7.5 minutes. Participants performed this process 6 times (3 hand tracking error conditions x 2 feedback states), resulting in a total of 45 minutes.

Additionally, participants completed the benchmark condition for Task 1 once between blocks of 3 repetitions, which took around 1 minute.

After Task 1, participants went through the training for Task 2, which took approximately 5 minutes on average. Then, completing Task 2 with 3 repetitions took around 9 minutes on average. Including the questionnaire completion, the time for each feedback state in Task 2 was approximately 11.5 minutes. With 2 feedback conditions for Task 2, the total time spent was 23 minutes. In summary, the total user study time including welcoming (5 min), training session for both tasks (3min + 5 min), no error benchmark condition (1 min for Task 1, 3 min for Task2), Task 1 (45 min), Task 2 (23 min) was around 85 minutes.
