# Supplementary material for: Before hands disappear: Effect of early warning visual feedback method for hand tracking failures in virtual reality
Source: PLoS One. 2025 Jun 10;20(6):e0323796. doi: 10.1371/journal.pone.0323796 (PMC12151392; doi:10.1371/journal.pone.0323796)
Supplement: S4 File — (PDF) [file pone.0323796.s004.pdf]

1 Detailed SUS Results

We found significant differences in the Friedman test results of Task 1 for all the SUS questions, as shown in Table 1. Then, in the Wilcoxon test results, we found significant differences for each hand tracking error, except Question 8 for the Out of Vision Hands error. The Wilcoxon test results for the Task 1 SUS questionnaire are given in Table 2.

Table 1: **Friedman Test results of Task 1 SUS data. We found significant differences in all questions.**

	**Friedman Test Result χ^2(5,18)**
**Q1**	χ^2 = 43.391, p <0.001
**Q2**	χ^2 = 39.499, p <0.001
**Q3**	χ^2 = 33.573, p <0.001
**Q4**	χ^2 = 22.27, p <0.001
**Q5**	χ^2 = 30.294, p <0.001
**Q6**	χ^2 = 32.677, p <0.001
**Q7**	χ^2 = 34.683, p <0.001
**Q8**	χ^2 = 19.882, p <0.001
**Q9**	χ^2 = 40.557, p <0.001
**Q10**	χ^2 = 28.257, p <0.001

Table 2: **Wilcoxon Test Results of the Task 1 SUS questionnaire data, differences between Feedback ON and OFF. There was no significant difference in Question 8 for the Out of Vision Hands error.**

**Question**	**Low Intensity Light**	**Out of Vision**	**Self Occlusion**
**Q1**	z = -3.22, p <0.001	z = -3.048, p <0.01	z = -3.33, p <0.001
**Q2**	z = -2.958, p <0.01	z = -2.7, p <0.01	z = -2.638, p <0.01
**Q3**	z = -2.954, p <0.01	z = -2.971, p <0.01	z = -2.958, p <0.01
**Q4**	z = -2.506, p <0.01	z = -2.066, p = 0.03	z = -2.047, p = 0.04
**Q5**	z = -2.994, p <0.01	z = -2.417, p = 0.01	z = -2.899, p <0.01
**Q6**	z = -2.85, p <0.01	z = -2.048, p = 0.04	z = -3.112, p <0.01
**Q7**	z = -2.994, p <0.01	z = -2.616, p <0.01	z = -2.84, p <0.01
**Q8**	z = -2.11, p = 0.03	z = -1.077, p = n.s.	z = -2.271, p = 0.02
**Q9**	z = -2.915, p <0.01	z = -3.37, p <0.001	z = -2.921, p <0.01
**Q10**	z = -2.292, p = 0.02	z = -2.481, p = 0.01	z = -2.719, p <0.01

The Wilcoxon results where we compared Feedback ON and OFF for the Task 2 SUS questionnaire revealed significant differences for all questions except Question 8. The test results are given in Table 3. When we looked at the SUS questions, we observed that participants preferred to use the feedback method more than without feedback (Q1). Further, they felt more confident with the feedback compared to without feedback (Q9). They also thought there were more inconsistencies when there was no feedback (Q6). Additionally, they thought the feedback method was easy to use (Q3), not necessarily complex (Q2), well integrated (Q5), did not require the support of a technical person to be able to use it (Q4), and easy to learn (Q7). They thought that they didn't need to learn a lot of things to use it (Q10).

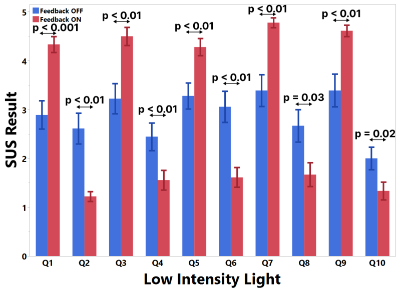

(a)

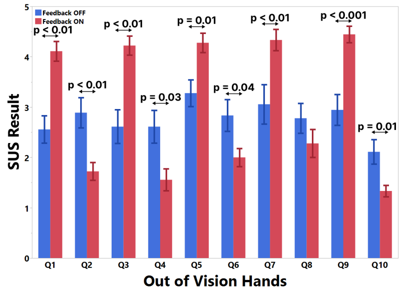

(b)

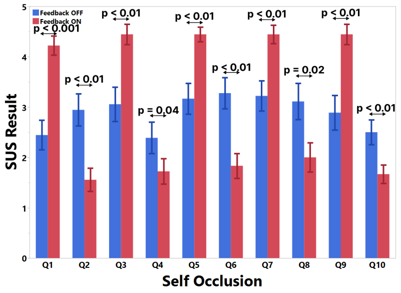

(c)

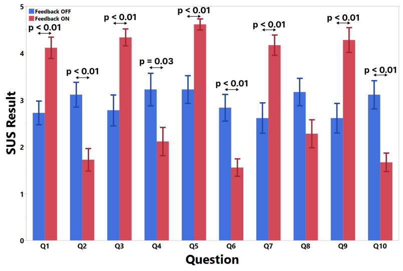

(d)

Figure 1: **Task 1 SUS Questionnaire Results across ten questions (Q1 - Q10) between Feedback ON and Feedback OFF for the hand tracking error (a) Low-Intensity Light, (b) Out of Vision Hands, and (c) Self Occlusion errors. The significant differences are indicated. (d) Task 2 SUS Questionnaire Results across ten questions (Q1 - Q10) for Feedback conditions: Feedback ON and Feedback OFF. The significant differences are indicated.**

Table 3: **Wilcoxon test results of the Task 2 SUS questionnaire data, showing differences between Feedback ON and OFF. We found no significant difference for Question 8.**

	Wilcoxon Test Result
**Q1**	z = -2.996, p <0.01
**Q2**	z = -2.898, p <0.01
**Q3**	z = -3.005, p <0.01
**Q4**	z = -2.162, p = 0.03
**Q5**	z = -3.108, p <0.01
**Q6**	z = -2.841, p <0.01
**Q7**	z = -3.028, p <0.01
**Q9**	z = -2.926, p <0.01
**Q10**	z = -2.774, p <0.01
