# Supplementary material for: Before hands disappear: Effect of early warning visual feedback method for hand tracking failures in virtual reality
Source: PLoS One. 2025 Jun 10;20(6):e0323796. doi: 10.1371/journal.pone.0323796 (PMC12151392; doi:10.1371/journal.pone.0323796)
Supplement: S5 File — (PDF) [file pone.0323796.s005.pdf]

Participant Comments

Each participant's individual comments, such as P1, are shared below. Since the comments section was not mandatory to fill, some participants skipped filling this section. These comments are indicated as "did not comment".

P1: "Feedback was useful"

P2: "[Early warning method] was very helpful in terms of notifying me when a [error] was about to happen and it helped mitigating it especially [middle of the] task."

P3: Did not comment.

P4: "The feedbacks were very useful and placed in a good place where they would be noticed easily yet not hide any of the activity that needed to be completed."

P5: Did not comment.

P6: Did not comment.

P7: Did not comment.

P8: "For the [low intensity light condition], I find it more useful when you are not aware of your surroundings (enclosed headset)".

P9: "I would prefer a floating panel for the feedback message, or text to speech system, or inform the warning with sound".

P10: Did not comment.

P11: "the system with feedback was easier to use and I would much rather have feed back while working with a system like this that is still new to people."

P12: "I believe the feedback system would help new VR users adapt faster to changes in vision and the environment. There is a visible need to more interactive tools to help the user navigate the virtual world in order to achieve a more satisfying experience."

P13: Did not comment.

P14: Did not comment.

P15: Did not comment.

P16: "I noticed that there is less feedback when the player achieve their goal. So if the system give stronger feedback information to the [participant], I think that [participants] can gain much more sense of achievement."

P17: Did not comment.

P18: "I think [early warning method is] a good feature to have to know the reasons why the hand motions may not be working. The user can adjust their movements depending on what the feedback says. The user can change its environment to solve the problem."
