# Supplementary material for: Before hands disappear: Effect of early warning visual feedback method for hand tracking failures in virtual reality
Source: PLoS One. 2025 Jun 10;20(6):e0323796. doi: 10.1371/journal.pone.0323796 (PMC12151392; doi:10.1371/journal.pone.0323796)
Supplement: Attachment — Submitted filename: Response to Reviewers.pdf [file pone.0323796.s006.pdf]

Monday, April 7, 2025

Dear Coordinator,

We thank you and both reviewers for their comments. We made the following changes based on the R1 review:

The Figure 1 added to text truly but it needs modification at all because it is one of the most important figures of the article. The steps are different in context and content so the colors of the boxes must be different. The steps must be connected to each other with arrows not lines. The size of the boxes must be smaller to have a better graphical design. The texts in the boxes must be about "what the authors done in that step?". All the texts should imply an activity i.e.:
- "Implementing Statistical Data Analysis Tests" not "Statistical Data Analysis Tests".
- "Inference the recommendations and write the conclusion" not "Conclusion and Recommendations".

* We grouped the boxes and applied a color-blind-friendly palette. Now, in Figure 1, green represents the literature review, blue task implementation, orange feedback design, pink user study and data analysis, and red discussion and conclusion.

* Each step now is connected to each other with arrows.

* We re-sized the boxes to have a better graphical design.

* We edited the text to emphasize an activity, as suggested by the reviewer.

We hope that these changes meet PLOS ONE’s publication criteria.

Kind regards

Mucahit Gemici
